# Top-down strategy synthesis of fluorinated graphdiyne for lithium ion battery†

**DOI:** 10.1039/c9ra05974a

**Published:** 2019-10-02

**Authors:** Huifang Kang, Yue Chen, Lanqing Xu, Yuda Lin, Qian Feng, Hurong Yao, Yongping Zheng

**Affiliations:** College of Physics and Energy, Fujian Normal University, Fujian Provincial Key Laboratory of Quantum Manipulation and New Energy Materials Fuzhou 350117 China zyp@fjnu.edu.cn; College of Photonic and Electronic Engineering, Fujian Normal University Fuzhou 350117 China lanqingxu@fjnu.edu.cn; Fujian Provincial Collaborative Innovation Center for Optoelectronic Semiconductors and Efficient Devices Xiamen 361005 China; Laboratory of Solid State Microstructures, Nanjing University Nanjing 210093 China

## Abstract

As a novel carbon allotrope, graphdiyne exhibits excellent electrochemical properties such as high specific capacities, outstanding rate performances, and long cycle lives. These properties are attributed to its sp- and sp^2^-hybridized bonding and a natural large pore structure. Doping with light elements is a facile way to improve the electrochemical performance of graphdiyne. Herein, we report the preparation of fluorine-doped graphdiyne by exposure to XeF_2_ under a mild temperature. Compared to pristine graphdiyne, the capacities are doubled. We obtained reversible capacities of fluorinated graphdiyne up to 1080 mA h g^−1^ after 600 cycles at a current density of 500 mA g^−1^. At a higher current density of 1000 mA g^−1^, it still retained a high specific capacity of 693 mA h g^−1^ after 1000 cycles. Using *in situ* quantitative nanomechanical probe atomic force microscopy, we further analyzed the surface morphologies and elastic modulus to understand the mechanism of the electrochemical improvement. The fluorinated graphdiyne elastic modulus is doubled in contrast to pristine graphdiyne. The performance improvements are attributed to the improvement in conductivity and enhancement of the mechanical properties.

## Abstract 2

Fluorine doped graphdiyne was synthesized by exposure to XeF_2_ under mild temperature. Owing to the enhanced conductivity and enhanced mechanical properties, the electrochemical performances are significantly improved.

## Graphical abstract

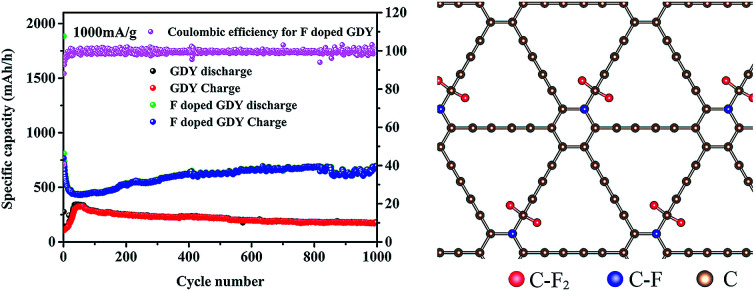

## 1 Introduction

There is a strong increasing demand for improving the capacity, rate performance, and life cycle of lithium-ion batteries (LIBS) to meet the requirements of electronics and the electric vehicle market.^1^ However, as a commercial LIBs anode material, the capacity of graphite is only 372 mA h g^−1^, and it is still far from supporting the demand for high energy density applications. Two-dimensional carbon-based materials such as graphene and graphdiyne (GDY) appear as attractive anode electrodes by virtue of their good conductivity, robust structure stability, high mechanical strength and efficient pathways for Li intercalation.^2,3^ As a new two-dimensional carbon allotrope, GDY possesses hybridized sp^2^ and sp carbon atoms.^4^ The diverse structural bondings enable colorful doping and structural modification, leading to new interesting properties including wide surface spacing, outstanding chemical stability and a unique electronic structure, making it be widely involved in energy, catalysis, optoelectronics and other fields.^5–9^ It has been reported that GDY can be used as a high-efficiency lithium storage material, exhibiting good chemical properties, high specific capacity, excellent rate performance and a long cycle.^10,11^ Li^+^ intercalation reaction in graphite occurs only at the edge and defect sites through the basal plane as LiC_6_.^12^ In contrast, GDY has 18-C hexagon pores and a layered structure which enable Li atoms to diffuse with high efficiency both in-plane and out-of-plane as LiC_3_. Theoretically, the predicted capacities for GDY reach to 744 mA h g^−1^.^13,14^ Experimentally, GDY was reported to possess high reversible capacities up to 520 mA h g^−1^ after 400 cycles at a current density of 500 mA g^−1^. The specific capacity is stable at 420 mA h g^−1^ even after 1000 cycles under a higher current density of 2 A g^−1^.^11^

Although GDY as an anode exhibits remarkable capacities and rate performance, the capacity of GDY is still not desirable. To meet the requirements in high power density applications, many efforts have been made to enhance the energy density and specific capacity of GDY-based LIBs, such as base-nanocrystallization and light elements doping methods.^15–19^ The doping method was considered as a simple and effective way to improve electrochemical performance in GDY. Two doping technologies have been reported, including *in situ* doping and mixing doping. As a bottom-up strategy, *in situ* doping is firstly doping hexaethylbenzene with H, Cl, F atoms and then synthesizing the doped GDY by cross-coupling reaction.^17–19^ Mixing doping is a top-down strategy by mixing GDY with other materials such as ammonium, boron oxide, ammonium fluoride and synthesizing under thermal annealing, heteroatom doped GDY can be obtained.^16,20^ The advantage of the bottom-up method is the doping elements can be doped in a uniform manner and form a large hexagonal pore to store up Li. The uniformity can extraordinarily improve the electrochemical performance.^17–19^ However, this method changes the GDY intrinsic structure, and so changes its intrinsic properties. Compared to the *in situ* doping method, the mixing method keeps the GDY intrinsic structure making it more economic and easy to implement. It has been reported that nitrogen doping can be an effective mixing doping way to increase the capacity and cycle performance.^16^ As we know, fluoridation can provide maximum charge polarization to enhance energy-related electrochemical activity and stability owing to fluorine’s higher electronegativity compared to nitrogen. Meanwhile, fluoridation can enhance the mechanical properties in carbon-based materials.^21,22^ The top-down strategy fluorine doping of GDY is expected to enhance its mechanical properties to improve the electrochemical performance as lithium storage materials.

Herein, we report facile fluorine doped graphdiyne synthesized by mixing GDY and XeF_2_ and reacting at a mild temperature. Electrochemical testing results show that the F doped GDY electrode exhibits excellent electrochemical properties, including higher reversible capacity and outstanding rate performance, compared to those of the pristine GDY electrode. We measured the elastic modulus using an AFM microscope through Peak Force QNM mode to analyse the mechanical changes after doping. As a result, the fluoridation can double the elastic modulus. The mechanical enhancement and conductivity improvements mean the F doped GDY achieves a highly improved specific capacity of 1080 mA h g^−1^ at a current density of 500 mA h g^−1^. After 1000 cycles, there is still a specific capacity of 693 mA h g^−1^ at a current of 1000 mA g^−1^.

## 2 Experimental section

### 2.1 Preparation of GDY films

Firstly, a piece of copper foil (2 × 8 cm^2^) was cleaned with 10 M hydrochloric acid (50 ml) for 5 minutes by sonication. Then it was further sonicated by water, ethanol and acetone sequentially for 5 minutes. The after-cleaned foil was dried under argon. Before reaction, the copper foil and pyridine (170 ml) were placed in a three-necked flask and heated at a temperature of 120 °C under an argon protected atmosphere. Then the temperature was steadily reduced to 80 °C. The hexakis[(trimethylsilyl)ethynyl]benzene (115 mg) was dissolved in a tetrahydrofuran solution (70 ml) at low temperature (0 °C), then tetrabutylammonium fluoride solution (TBAF) (1.8 ml) was added into the solution. The mixture was stirred for 15 minutes and the color of the solution gradually turned purple after adding TBAF. The reaction mixture was diluted with ethyl acetate, and washed with saturated sodium chloride solution sequentially. To remove the additional water, the solution was dried with anhydrous sodium sulfate. After evaporation *in vacuo*, the residue was dissolved dropwise into the mixed solution containing pyridine. The dropwise process lasts for 8 hours. Then the solution was reacted for more than 48 h. The entire synthesis process lasted approximately 3 days. The obtained samples were washed with hot (60 °C) dimethylformamide (DMF), acetone and ethanol, respectively. Finally, a GDY film was obtained by vacuum drying at 80 °C for 12 h.

### 2.2 Synthesis of fluorine-doped GDY films

The synthesized GDY (2 × 8 cm^2^) was cut into several pieces (2 × 1.3 cm^2^) and placed in a 50 ml argon-filled polyvinyl chloride reactor along with a quantity of XeF_2_. It was heated at 180 °C for 12 h, then cooled down to room temperature. In order to assemble a coin cell, the synthesized F doped GDY film was carefully scraped off one side to expose the copper base as binder. Then it was washed with acetone and ethanol in turn, and dried at 80 °C for 12 h in a vacuum.

### 2.3 Characterization

The surface morphologies of F doped GDY and GDY were observed using a scanning electron microscope (SEM, JSM-7500F, Japan). Raman spectra were obtained using a LABRAM-HRmicro-Raman system (Longjumeau, Paris, France). The chemical compositions of the samples were measured by X-ray photo-electron spectroscopy (XPS, ESCALAB 250, VG, USA), and also confirmed by Fourier Transform Infrared Spectroscopy (FTIR, Thermo-Fisher Nicolet iS50). The surface environments of the samples were observed by atomic force microscopy (AFM). Modulus measurements were performed using an AFM microscope (Dimension icon, Bruker) through Peak Force QNM mode with ScanAsyst™ in an argon protected atmosphere.

### 2.4 Electrochemical measurements

Electrochemical experiments were performed in a CR2025 coin cell. No initial binder was needed because the copper foil can be used as an electrode directly. All battery components were assembled in standard glove boxes. A lithium metal foil was used as the counter electrode and reference electrode. The electrolyte was prepared by dissolving 1 M LiPF_6_ into a 1 : 1 (v/v) mixture of ethylene carbonate (EC) and dimethyl carbonate (DMC). The galvano charge and discharge cycle performance was measured by the LAND CT2001A battery test system. The cycle performance of the battery was tested at 500 mA g^−1^ and 1000 mA g^−1^; the rate performance was tested at different current densities from 50 mA g^−1^ to 1500 mA g^−1^. In the electrochemical workstation (CHI 660C), cyclic voltammetry was carried out at a voltage ranging from 0.01 V to 3 V with a scanning rate of 0.1 mV s^−1^.

## 3 Results and discussion

**Fig. 1:**
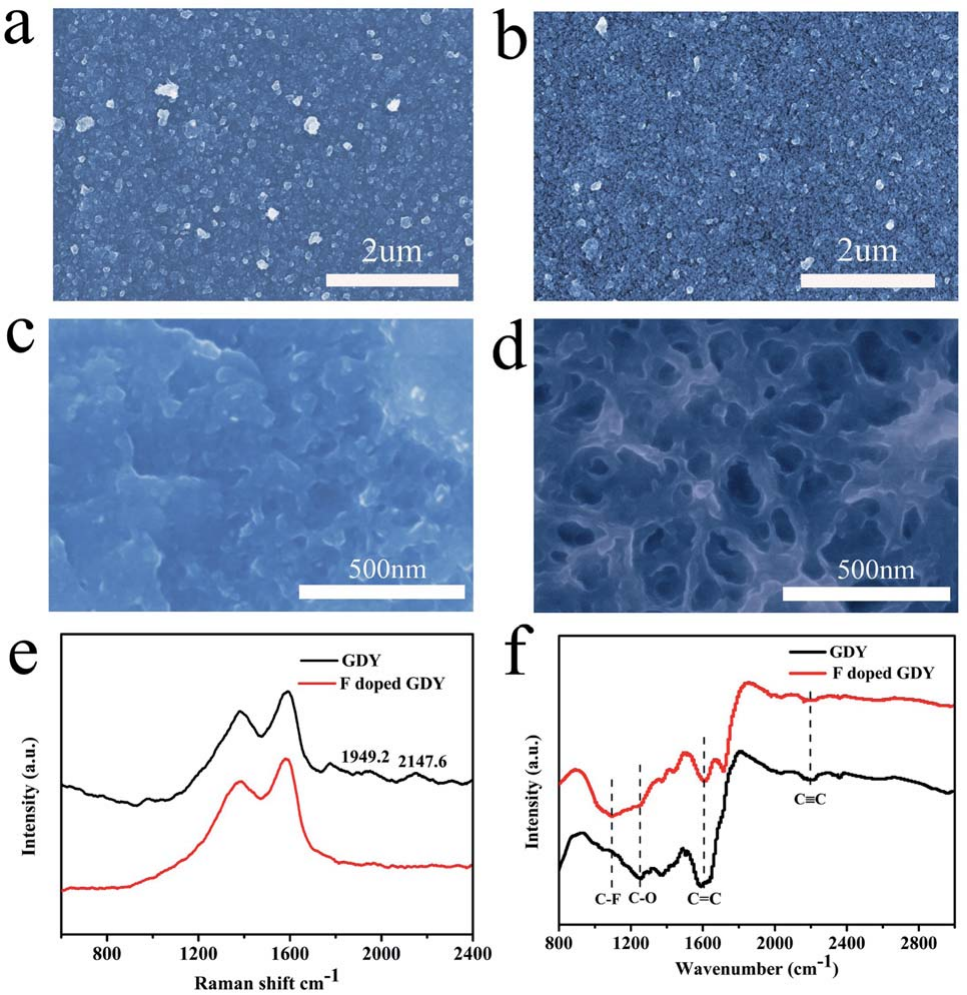
(a) SEM image morphology of GDY film grown on the surface of copper foil. (b) SEM morphology of the F doped GDY film. High-magnification SEM of (c) GDY and (d) F doped GDY. (e) Raman spectra of GDY and F doped GDY. (f) FTIR spectra of GDY and F doped GDY film on copper foil.

The morphologies of the membrane before and after fluoridation were characterized by SEM. As shown in Fig. 1a, the as-prepared GDY film is uniform, indicating that the GDY film was continuously grown on the copper foil. The morphology of F doped GDY shown in Fig. 1b is similar to that of the GDY film, which means the original structural layout was well maintained during the F-doping process (ESI Fig. S1 and Table S1†). TEM images further demonstrated the uniform and continuous films of GDY and F doped GDY with multilayer structure (ESI Fig. S2†). Fig. 1c and d are high-magnification SEM images of GDY and F doped GDY. After F doping, the pore size of the surface is significantly increased. The thicknesses of the GDY and F doped GDY films are approximately 678 nm and 610 nm, respectively (ESI Fig. S3†). The Raman spectra of the GDY and F doped GDY films are shown in Fig. 1e. It is clear to observe there are four main peaks on both samples. The D band of GDY is located at 1379.6 cm^−1^, while the D band of F doped GDY slightly shifts to 1383.5 cm^−1^, mainly due to structural defects. The G band is located at 1590.2 cm^−1^ for GDY and 1585.5 cm^−1^ for F doped GDY. These peaks correspond to the first-order scattering of the E_2g_ mode of in-phase stretching vibrations and the breathing vibration of sp^2^-hybridized carbon domains in aromatic rings, respectively. The ratios of the intensities of the D and G bands (*I*_D_/*I*_G_) for GDY and F doped GDY are 0.8 and 0.84, respectively, which indicate both of them are highly ordered. A lower value of *I*_D_/*I*_G_ in the F doped GDY case corresponds to more defects and disorder after F doping.^4^ Two other peaks, in the GDY sample the peaks at 2147.6 cm^−1^ and 1949.2 cm^−1^, should be the vibration in the acetylenic bond (–C≡C–C≡C–).^4,16^ After fluoridation, the alkyne peak becomes weak. The structures of GDY and F doped GDY were further investigated using FTIR as shown in Fig. 1f. The peak of 1500–1650 cm^−1^ is believed to originate from the skeleton vibration of the aromatic ring. The band at 1251 cm^−1^ is assigned to the C–O stretching.^23^ The weak band observed at *ca.* 2200 cm^−1^ is attributed to the acetylene band. The results of FTIR are consistent with previous reports.^17,18^ Due to the C–F stretching vibration and the aromatic bending vibration, an extra peak at *ca.* 1098 cm^−1^ can be observed in the F doped GDY circumstance.^24^

**Fig. 2:**
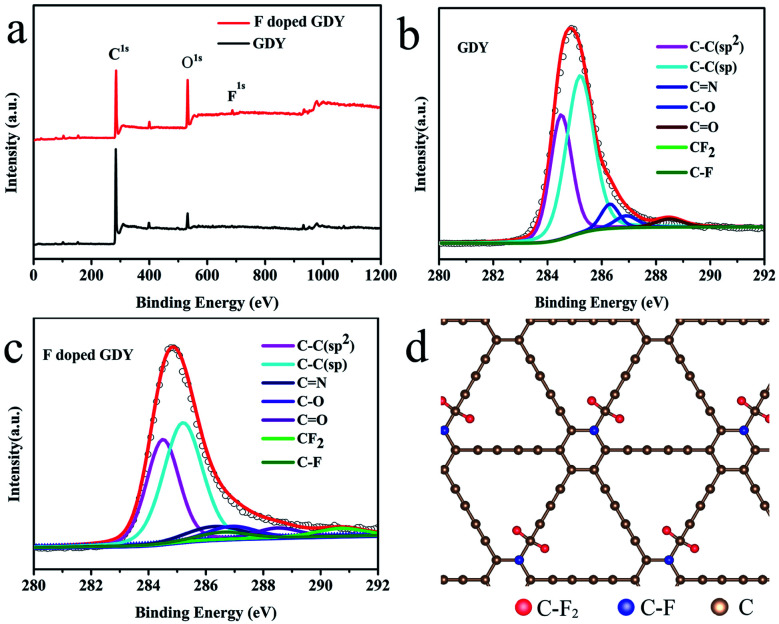
(a) XPS survey of GDY and F doped GDY over a wide range of binding energies (0–1200 eV). (b) C 1s spectrum of GDY. (c) C 1s spectrum of F doped GDY. (d) Schematic illustration of F doped GDY structure, red atoms are F bonded with C at ring sites formed as C–F_2_, blue atoms are F bonded with C at chain sites formed as C–F.

To explore the chemical environment variations after fluoridation, further XPS characterizations were performed. As shown in Fig. 2a, XPS full range spectroscopy of GDY shows that only the C 1s and O 1s peaks appear at 284.8 and 532.0 eV, respectively. The O atom was introduced by oxidization during the synthesizing procedure. For the F doped GDY sample, the F 1s peak at 688.0 eV and F Auger peak at 832.5 eV are seen clearly, showing that the F atoms were doped successfully.^25^Fig. 2b gives the typical C 1s XPS spectrum of GDY. After subtraction of the Shirley background, followed by fitting with a mixture function of Lorentzian and Gaussian, we could deconvolve the C 1s peak into four main sub-peaks at 284.5, 285.2, 286.9 and 288.5 eV, which can be attributed to C–C (sp^2^), C–C (sp), C–O and C=O bonds, respectively.^4,26^ The chemical contents are list in Table 1. The area ratio of sp/sp^2^ is 1.59, which indicates that the diacetyl bond has a better linkage to the benzene ring.^4^ As for F doped GDY, in addition to the above four peaks mentioned, there are two more peaks at 286.6 and 290.7 eV (Fig. 2c), which correspond to the C–F bond and C–F_2_ bond respectively.^20,27^ The area ratio of sp/sp^2^ reduces to 1.35, indicating that C–F_2_ bonds are formed by F atoms bonding with the diacetyl link, resulting in a 3.3% C–F_2_ coverage as shown in Table 1. Further more there is a 3.7% C–F bond coverage, whereas the content of C=C bonds reduces from 34% to 32%, which shows that the F atoms are also likely to bond with benzene rings, leading to a conversion from sp^2^ to sp^3^ bonding structure on the membrane. A schematic illustration of the fluoridation structure is shown in Fig. 2d.

**Table 1:** The chemical compositions of GDY and F doped GDY and deconvolved O and F 1s spectra

Chemical groups	C=C (sp^2^)	C–C (sp)	C–N	C–O, C=O	C–F	C–F_2_
Location (eV)	284.5	285.2	286.3	286.9, 288.5	286.6	290.7
GDY (%)	34.0	54.3	5.4	6.3	0	0
F doped GDY (%)	32.4	43.8	6.9	9.9	3.7	3.3

**Fig. 3:**
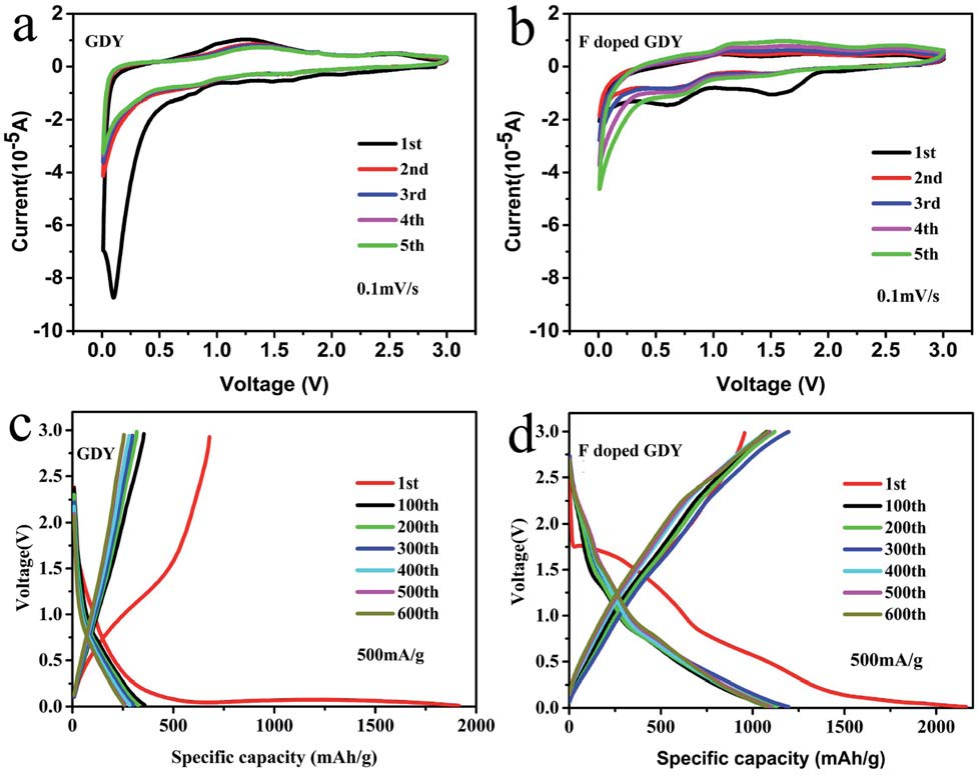
(a and b) Cyclic voltammetry curves of the GDY and F doped GDY based electrode, scan rate is 0.1 mV s^−1^. (c and d) Galvanostatic charge/discharge profiles of GDY and F doped GDY electrodes at a current density 500 mA g^−1^, recorded between 0.01 mV and 3 V.

**Fig. 4:**
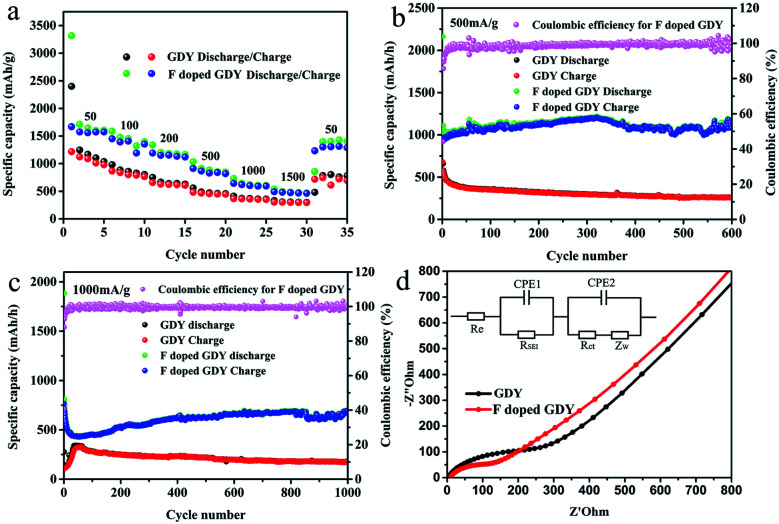
(a) Different rate performances of F doped GDY and GDY electrodes at varied current densities. (b) Cycle performance of F doped GDY and GDY electrodes under 500 mA g^−1^. (c) Cycle performance of F doped GDY and GDY electrodes under 1000 mA g^−1^. (d) Nyquist plots of GDY and F doped GDY electrodes after 50 cycles under 500 mA g^−1^, and the inset is the equivalent circuit. *R*_e_ represents the electrolyte resistance. *R*_SEI_ is the resistance of SEI and CPE1 is its capacitance. *R*_ct_ is a charge transfer resistor. CPE2 represents a double layer capacitor. *Z*_W_ is the impedance of Warburg.

The electrochemical properties of F doped GDY were tested using a CR2025 coin-type half cell. The copper foils are directly used as electrodes. However, the copper foils will be dissolved during the reaction process according to the synthetic mechanism of GDY. The mass of active materials was carefully calculated by SEM cross-section characterization and described in ESI Fig. S3 and Table S2.† The cyclic voltammograms (CV) of GDY and F doped GDY are shown in Fig. 3a and b at a scan rate of 0.1 mV s^−1^. Both of them exhibit an irreversible CV band during the first cathodic scanning. There is no clear reduction in the GDY case consistent with a previous report.^11^ However, F doped GDY showed a significant reduction peak at 0.62 V, an oxidation peak appeared at 0.95 V and an extra reduction peak appeared at 1.5 V during the first cathode scan. The previous XPS analysis showed that F doped GDY has C–F and C–F_2_ bonds after fluoridation. The two reduction peaks correspond to the different C–F bonding reductions. After the first cathode scan, the 1.5 V peak disappeared due to decomposition of the electrolyte and the formation of a solid electrolyte interface (SEI) film.^11,16,26^ From the second cycle, the CV curves are essentially coincident. This indicates that F doped GDY has high reversible charging and discharging behaviors. Fig. 3c and d show the charge and discharge curves for GDY and F doped GDY, respectively. The charge and discharge curves for 1, 100, 200, 300, 400, 500, and 600 cycles were recorded separately. The specific capacities of the F doped GDY first discharge/charge are 2163 mA h g^−1^ and 955 mA h g^−1^, respectively. Compared to GDY, the initial coulombic efficiency, cycle life and reversible capacity of F doped GDY are significantly enhanced. The coulombic efficiency of the original GDY (35%) (ESI Fig. S4†) was increased to 44% in the F doped GDY situation (seen in Fig. 4b). The improvement demonstrates that the doping of F can effectively suppress the decomposition of the electrolyte and the reaction on the surface side to form an SEI film on the F doped GDY electrode. As show in Fig. 3d, the capacity of the 300th discharge was reduced to 1195 mA h g^−1^ compared to the first discharge capacity. The reduction can be ascribed to the adsorption of strong lithium ions at specific active sites such C–F_2_ GDY chain sites. After the 600th cycle, the capacity is still above 1080 mA h g^−1^, indicating that the F doped GDY material has good capacity retention which is superior to N doping GDY.^16^

It can be seen from Fig. 4a that F doped GDY exhibits a superior rate performance to GDY within a current density range of 50 to 1500 mA g^−1^. The reversible capacity is about 1600 mA h g^−1^ at a current density of 50 mA g^−1^. Even at a high current density of 1500 mA g^−1^, the reversible capacity is still above 500 mA h g^−1^. In contrast, the specific capacity of the GDY electrode is 1100 mA h g^−1^ at a current density of 50 mA g^−1^ and 300 mA h g^−1^ at a current density of 1500 mA g^−1^, respectively. When the current density is reset to 50 mA g^−1^, F doped GDY can still maintain a high reversible capacity of 1400 mA h g^−1^, in contrast to a significant shrinking to 761 mA h g^−1^ in the GDY case. The excellent rate performance of F doped GDY is suggested to originate from the electronegativity of the F atoms. F doping is an effective method to improve the kinetics of lithium diffusion and migration. These phenomena can be better observed in Fig. 4b and c. At 500 mA g^−1^ current density, the electrode capacity of F doped GDY shows a slightly upward trend and reaches to about 1080 mA h g^−1^ after 600 cycles. On the contrary, the reversible capacity of GDY gradually decreases to about 300 mA h g^−1^ after 600 cycles, which means that F doped GDY has better stability than that of pristine GDY. As shown in Fig. 4c, when the current density is increased to 1000 mA g^−1^, the reversible capacity of F doped GDY reaches 693 mA h g^−1^ and continues to rise even after 1000 cycles, whereas the reversible capacity of GDY reduces to approximately 200 mA h g^−1^ after 1000 cycles. The optimized GDY and XeF_2_ mass ratio is 1 : 16 (ESI Fig. S5†). These phenomena show that F doped GDY has a more stable and larger reversible capacity than that of GDY. It has been reported that the defects on the N-doped graphene sheets provide more Li^+^ storage active sites during the charging/discharging process.^28^ The adsorption energy of Li^+^ is larger and the energy barrier for lithium permeability is lower around the defects, therefore the introduction of F atoms can increase electron conductivity and generate more hereto atom defects and electrochemically active sites on the GDY membrane. Thus higher capacity was achieved. Moreover, fluorine doping benefits minimization of surface side reactions and formation of a stable interface, thereby improving the electrochemical stability and the reversible capacity of the F doped GDY electrode during the cycling process.

To penetrate more into the depth of the Li storage process after F doping, electrochemical impedance spectroscopy (EIS) measurements were performed. The testing frequency ranges from 0.01 Hz to 100 kHz, and the results after 50 cycles are illustrated in Fig. 4d. The Nyquist diagram shows that F doped GDY has a semicircle diameter smaller than that of the GDY electrode in the high intermediate frequency region, which means that the contact resistance and charge transfer impedance are reduced by F-doping. Different reaction kinetics between GDY and F doped GDY samples were also investigated. The EIS spectra were fitted using the circuit as shown in the inset of Fig. 4d. The electrolyte resistance *R*_e_ of GDY and F doped GDY obtained by fitting with the Nyquist plot are listed in Table 2. Both GDY and F doped GDY exhibit similar *R*_e_ (6.28 Ω and 6.14 Ω, respectively). However, the SEI resistance of the F doped GDY electrode (*R*_SEI_ = 48.88 Ω) is much lower than that of GDY (*R*_SEI_ = 123.10 Ω), showing that the doping of the F atoms can significantly suppress the SEI resistance. The charge transfer resistance of the F doped GDY electrode (*R*_ct_ = 90.0 Ω) is also much smaller than that of the GDY electrode (*R*_ct_ = 257.80 Ω). Owing to F-doping, reduced *R*_SEI_ and *R*_ct_ are achieved, thus higher conductivity can be expected.

**Table 2:** Kinetic parameters of GDY and F doped GDY after 50 cycles under 500 mA g^−1^

Samples	GDY	F doped GDY
*R*_e_ (Ω)	6.28	6.14
*R*_ct_ (Ω)	257.80	90.0
*R*_SEI_ (Ω)	123.10	48.88

**Fig. 5:**
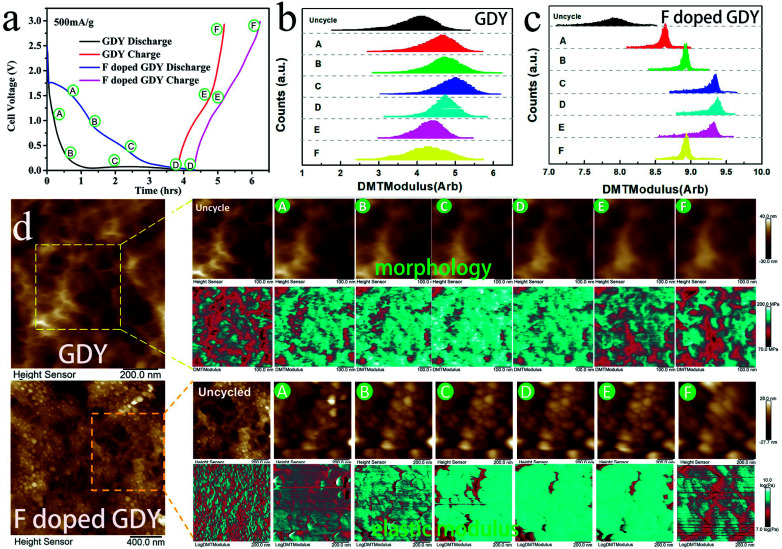
(a) Galvanostatic cycling of GDY and F doped GDY at 500 mA g^−1^. A to D correspond to the intercalation process and D to F correspond to the deintercalation process. Area statistics of elastic modulus variations in different states during discharge/charge of (b) GDY and (c) F doped GDY. (d) *In situ* Kelvin probe atomic force microscope record of morphology and elastic modulus phase in different chemical states during discharge/charge.

In addition, the high conductivity can promote the electrochemical properties. There have been reports that mechanical enhancement can also improve electrochemical properties previously.^29–31^ Fluorination is an effective method to improve the thermal and mechanical properties within carbon materials.^32–34^ In order to investigate the mechanical changes of GDY and F doped GDY in the electrochemical process, an *in situ* quantitative nanomechanical (QNM) atomic force microscope was used to the measure the elastic modulus variations during the first discharge/charge cycle. A commercial silicon nitride tip (RTESPA, cantilever resonance frequency of 200 kHz and nominal elastic constant of 40 N m^−1^) with a typical apex radius of 10 nm was used. Spring constants and resonance frequencies of the cantilevers were both acquired *via* the thermal-tuning in software. The deflection sensitivity and tip radius were calibrated against standard samples before and after experiments. Maps of the reduced elastic modulus (*E*_r_) were fitted from the force–distance curves at each pixel through the Derjaguin–Muller–Toporov model,^35^1
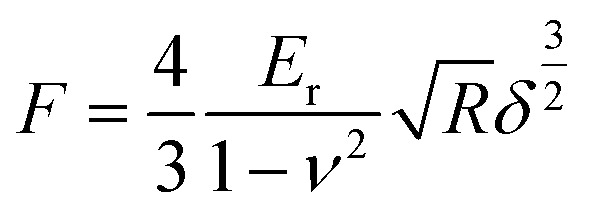
where *F* and *δ* are the force and indentation which can be obtained from the force curve, respectively. *R* is radius of the tip, *ν* is the Poisson’s ratio. Here, we chose *ν* = 0.2 to calculate the reduced elastic modulus *E*_r_ for the qualitative analysis. All parameters in the formula were dimensionless to enable a visual comparison of the *E*_r_ value of the GDY and F doped GDY samples. Galvanostatic cycling was performed using a LAND CT2001A battery test system at 500 mA g^−1^ in a voltage range of 0.01–3 V (Fig. 5a). During the procedure of intercalation (from A to D) and deintercalation (from D to F) of lithium, different chemical states including morphologies and elastic modulus phases were recorded and displayed in Fig. 5d. It can be clearly seen from Fig. 5d that the morphologies of both GDY and F doped GDY blur gradually, which can be ascribed the SEI film formation. The elastic modulus are increased during the intercalation process both in GDY and F doped GDY, whereas in reverse during the deintercalation process. To quantize the mechanical variations of intercalation and deintercalation, we calculated the area statistics of the elastic modulus in the dashed square for different states and present them in Fig. 5b and c for comparison. In the case of GDY, the uncycle elastic modulus peak is 4.12. It then increases to 5.03 when the Li^+^ intercalates into GDY and decreases to 4.29 when it deintercalates. As previous reported, Li^+^ atoms captured by 18-C hexagon prefer to adsorb on the other side to avoid the repulsion with Li adsorbed over the 6-C hexagon.^13,14^ The aggregation of Li^+^ atoms contribute to enhance the mechanical properties. After deintercalation, the elastic modulus enlarges slightly, owing to the residual Li^+^ atoms bonding with O defects. The variations in elastic modulus are similar in the F doped GDY case, increasing from 7.92 to 9.57 and then decreasing to 8.93 during discharge/charge. Different to that of GDY, the residual Li^+^ atoms bond not only with O defects but also with C–F bonds, leading to cross-references elastic variation greater than GDY. With a comparison of Fig. 5b and c, we can see that fluoridation almost doubled the elastic modulus of GDY from 4.12 to 7.92. These remarkable mechanical changes contribute to improved reversible capacity and electrochemical stability in F doped GDY during cycling.^30^

## 4 Conclusions

In summary, in this work F doped GDY are facilely synthesized by a mixing GDY and XeF_2_ reaction at a mild temperature. Various techniques such as SEM, Raman, FTIR and XPS were applied to investigate the morphology changes and electrochemical properties after fluorine doping. The chemical structure results show that F atoms bond with C on both benzene rings and diine links. Both samples can used as a negative electrode in lithium-ion batteries to evaluate their lithium storage ability. Compared with pristine GDY, F doped GDY has a better electrochemical performance, such as high specific capacity, excellent rate performance and good cycle life. The current density is 500 mA g^−1^ and the specific capacity is 1080 mA h g^−1^. After 1000 cycles, there is still a specific capacity of 693 mA h g^−1^ at a current of 1000 mA g^−1^. Thus, the electrochemical performance is significantly improved after fluorine doping. The introduction of F atoms produces more atom defects and electrochemically active sites contributing to the enhancement in capacity. The remarkable mechanical enhancement within F doped GDY contributes to improving its reversible capacity and electrochemical stability compared to GDY during cycling. Our results show that F doped GDY will serve as a promising anode material for lithium-ion batteries.

## Conflicts of interest

There are no conflicts to declare.

## Supplementary Material

RA-009-C9RA05974A-s001

## References

[cit1] Turcheniuk K., Bondarev D., Singhal V., Yushin G. (2018). Nature.

[cit2] Yao F., Pham D. T., Lee Y. H. (2015). ChemSusChem.

[cit3] Wang G., Shen X., Yao J., Park J. (2009). Carbon.

[cit4] Li G., Li Y., Liu H., Guo Y., Li Y., Zhu D. (2010). Chem. Commun..

[cit5] Zhuang X., Mao L., Li Y. (2017). Electrochem. Commun..

[cit6] Jia Z., Li Y., Zuo Z., Liu H., Huang C., Li Y. (2017). Acc. Chem. Res..

[cit7] Xiao J., Shi J., Liu H., Xu Y., Lv S., Luo Y., Li D., Meng Q., Li Y. (2015). Adv. Energy Mater..

[cit8] Jin Z., Zhou Q., Chen Y., Mao P., Li H., Liu H., Wang J., Li Y. (2016). Adv. Mater..

[cit9] Huang C., Li Y., Wang N., Xue Y., Zuo Z., Liu H., Li Y. (2018). Chem. Rev..

[cit10] Du H., Yang H., Huang C., He J., Liu H., Li Y. (2016). Nano Energy.

[cit11] Huang C., Zhang S., Liu H., Li Y., Cui G., Li Y. (2015). Nano Energy.

[cit12] Jungblut B., Hoinkis E. (1989). Phys. Rev. B: Condens. Matter Mater. Phys..

[cit13] Sun C., Searles D. J. (2012). J. Phys. Chem. C.

[cit14] Zhang H., Xia Y., Bu H., Wang X., Zhang M., Luo Y., Zhao M. (2013). J. Appl. Phys..

[cit15] Shang H., Zuo Z., Yu L., Wang F., He F., Li Y. (2018). Adv. Mater..

[cit16] Zhang S., Du H., He J., Huang C., Liu H., Cui G., Li Y. (2016). ACS Appl. Mater. Interfaces.

[cit17] He J., Wang N., Cui Z., Du H., Fu L., Huang C., Yang Z., Shen X., Yi Y., Tu Z. (2017). et al.. Nat. Commun..

[cit18] Wang N., He J., Tu Z., Yang Z., Zhao F., Li X., Huang C., Wang K., Jiu T., Yi Y. (2017). et al.. Angew. Chem..

[cit19] He J., Wang N., Yang Z., Shen X., Wang K., Huang C., Yi Y., Tu Z., Li Y. (2018). Energy Environ. Sci..

[cit20] Zhang S., Cai Y., He H., Zhang Y., Liu R., Cao H., Wang M., Liu J., Zhang G., Li Y. (2016). et al.. J. Mater. Chem. A.

[cit21] Shofner M. L., Khabashesku V. N., Barrera E. V. (2006). Chem. Mater..

[cit22] Abdalla M., Dean D., Theodore M., Fielding J., Nyairo E., Price G. (2010). Polymer.

[cit23] Li Y., Guo C., Li J., Liao W., Li Z., Zhang J., Chen C. (2017). Carbon.

[cit24] Song Y., Li X., Yang Z., Wang J., Liu C., Xie C., Wang H., Huang C. (2019). Chem. Commun..

[cit25] Feng Q., Xiao W., Liu Y., Zheng Y., Lin Y., Li J., Ye Q., Huang Z. (2018). Materials.

[cit26] Zhang S., Liu H., Huang C., Cui G., Li Y. (2015). Chem. Commun..

[cit27] Ross G., Watts J., Hill M., Morrissey P. (2000). Polymer.

[cit28] Reddy A. L. M., Srivastava A., Gowda S. R., Gullapalli H., Dubey M., Ajayan P. M. (2010). ACS Nano.

[cit29] Zhao K., Wang W. L., Gregoire J., Pharr M., Suo Z., Vlassak J. J., Kaxiras E. (2011). Nano Lett..

[cit30] Zhao K., Pharr M., Cai S., Vlassak J. J., Suo Z. (2011). J. Am. Ceram. Soc..

[cit31] Zhao K., Tritsaris G. A., Pharr M., Wang W. L., Okeke O., Suo Z., Vlassak J. J., Kaxiras E. (2012). Nano Lett..

[cit32] Davami K., Jiang Y., Lin C., Cortes J., Robinson J. T., Turner K. T., Bargatin I. (2016). RSC Adv..

[cit33] Wang X., Wu P. (2018). ACS Appl. Mater. Interfaces.

[cit34] Nair R. R., Ren W., Jalil R., Riaz I., Kravets V. G., Britnell L., Blake P., Schedin F., Mayorov A. S., Yuan S. (2010). et al.. Small.

[cit35] Meng X., Zhang H., Song J., Fan X., Sun L., Xie H. (2017). Nat. Commun..

