# Supplementary material for: Top-down strategy synthesis of fluorinated graphdiyne for lithium ion battery
Source: RSC Adv. 2019 Oct 2;9(54):31406–12. doi: 10.1039/c9ra05974a (PMC9072409; doi:10.1039/c9ra05974a)
Supplement: RA-009-C9RA05974A-s001 [file RA-009-C9RA05974A-s001.pdf]

Electronic Supplementary Material (ESI) for RSC Advances.
This journal is © The Royal Society of Chemistry 2019

# Support Information

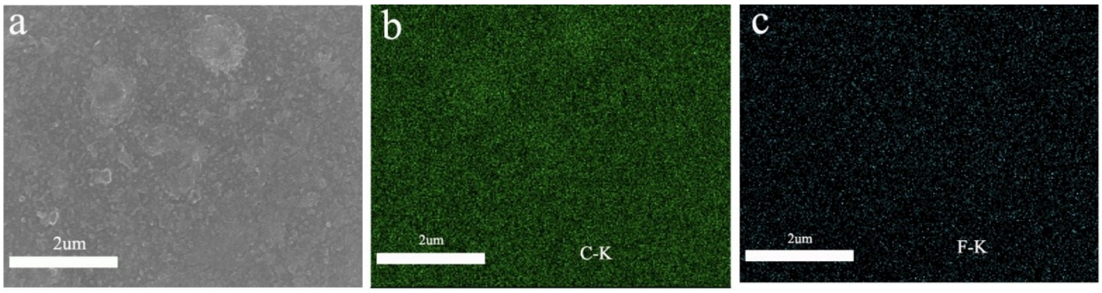


Fig. S1. SEM and EDS elemental mapping images of the F doped GDY film.

Table S1. Elemental content of F doped GDY from EDX.

| Element | Weight ratio (%) | Atom ratio (%) |
|---|---|---|
| C K | 82.26 | 88.00 |
| F K | 17.74 | 12.00 |
| total | 100.00 | 100.00 |

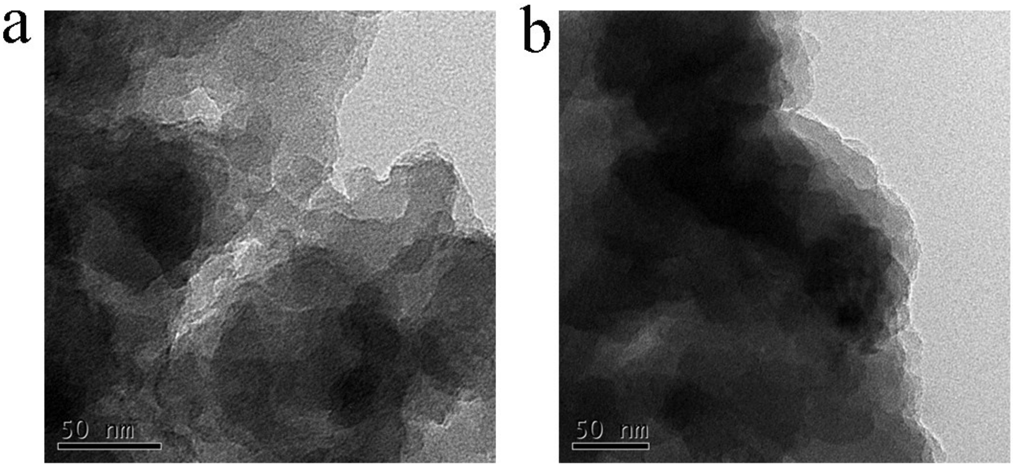


Fig. S2. TEM image, a is GDY, and b is F doped GDY.

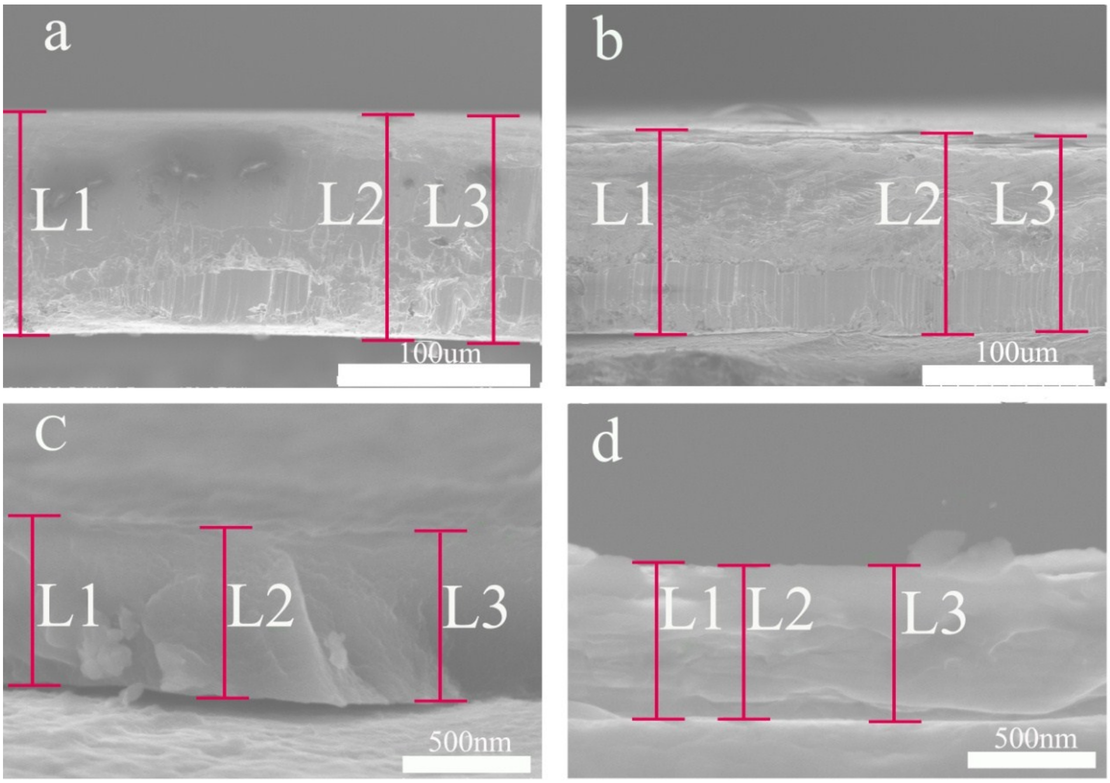


Fig. S3. (a) cross-sectional SEM images of (a) Cu, (b) Cu when GDY is synthesized, (C) GDY and (d) F doped GDY.

Table. S2 shows the thickness of the GDY and F doped GDY films are 678 nm and 610 nm, respectively. The copper consumption of the synthetic GDY process was 78 nm calculate from SEM cross-section. The density of GDY and F doped GDY are 1323.9 and 2386.6 mg/cm$^3$, respectively. The electrodes of GDY and F doped GDY have a diameter of 1.2 cm. The weight of GDY and F doped GDY were calculated by $m = \rho V$, list in the Table S2.

Table S2. The thickness and weight of the sample.

| | L1 | L2 | L3 | Average | Single electrodes weight |
|---|---|---|---|---|---|
| Cu | 115 um | 117 um | 116 um | 116 .0um | - |
| Cu+GDY | 118 um | 117 um | 115 um | 116.6 um | - |
| GDY | 680 nm | 674 nm | 680 nm | 678.0 nm | 0.10 mg |
| F doped GDY | 615 nm | 605 nm | 610nm | 610.0nm | 0.16 mg |

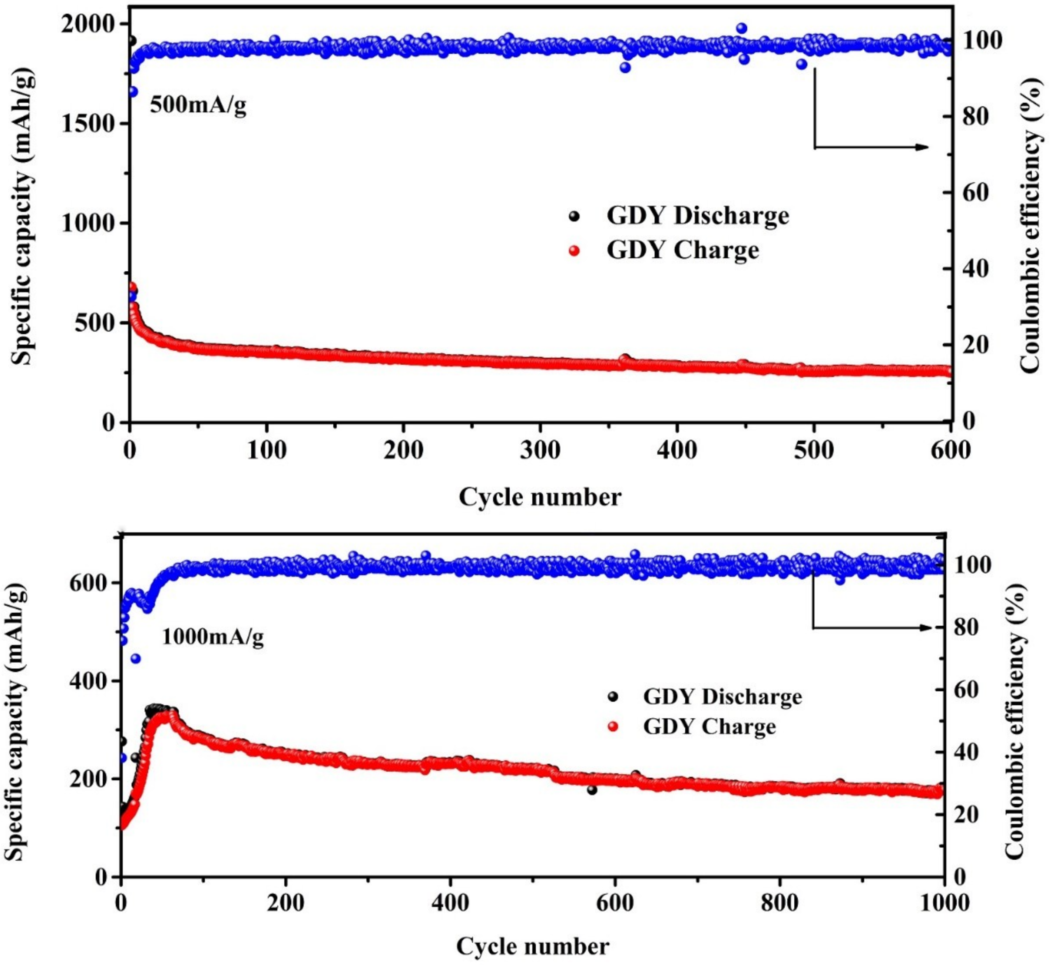


Fig. S4. The coulombic efficiency of GDY at a current density of 500 mA/g and 1000 mA/g.

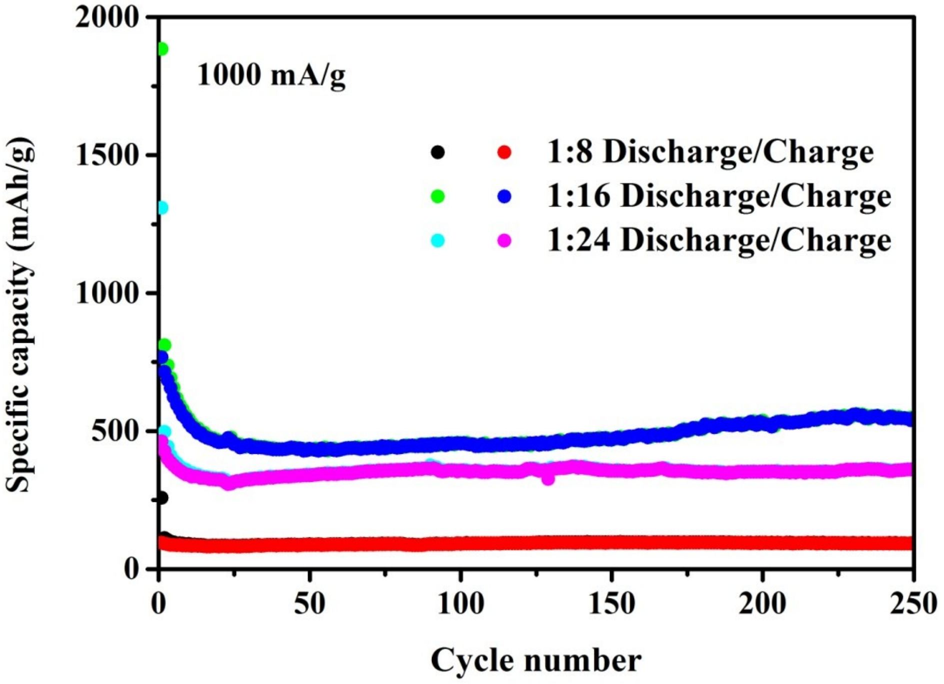


Fig. S5.Cycling performance of F-GDY under different concentrations of fluorine doped .
